# New York Letters

**Published:** 1882-11

**Authors:** 


### Domestic Correspondence.

ARTICLE XI.

TO THE EDITORS OF THE CHICAGO MEDICAL JOURNAL AND EXAMINER:

The most notable movement in the medical world here, is just now the development of post-graduate courses, and of opportunities for clinical study outside of the colleges. Heretofore a very large part of all clinical teaching has been done at Bellevue and Charity Hospitals, and in the dispensaries or out-door departments attached to the three larger colleges. The ambition now is to open up New York's other medical resources, and let those who come here to study, know that they can learn a great many things ouside of college curriculums. We have now two Post-graduate Medical Colleges. One is composed largely of the old clinical staff of the University College. It opens soon, and I am told, has promise of a good attendance.

The second college is a later organization, and is called the New York Policlinic. It announces that only clinical lectures will be given upon various medical and surgical branches. The faculty is quite large and includes some very good men. Dr. G. R. Leaminu is president, Dr. John A. Wyette, Dean and Professor of Surgery. The supply of Professors is abundant, there being two on Gynecology and Obstetrics, also two on Ophthalmology, Surgery and Dermatology. Neither instructor wants to be an Adjunct, and so harmony is secured by making both full fledged professors. The college promises well, and it is hard to say now, which offers the better opportunities.

In addition to these colleges, there is a movement to utilize the clinical material of the dispensaries. This is begun by the

North-Eastern Dispensary, one of the largest, and in prospect, wealthiest in the city. Clinical courses to be given in gynecology, by Dr. J. H. Gunning; Eye, Ear, and Throat Diseases, by Dr. Simon Baruch; Skin Diseases, by Dr. Wooster Beach; Nervous Diseases, by Dr. Chas. L. Dana; Heart and Lung Diseases, by Dr. D. M. Camman.

Society work began this Fall with a session of the Medico-Legal Society. Dr. Greene M. Hammond read a paper upon "Death by Hanging," in which he aimed to show that such mode of death is painless and prompt, when the hanging is properly done. The neck is rarely broken, but death takes place by suffocation. The convulsions and twitchings often seen, are asserted to be reflex, and not to indicate suffering.

A stormy debate followed, upon the subject of the Treasurer's action in not paying certain bills.

At the first meeting of the County Medical Society, this Fall, Dr. W. F. Miltendorf read a paper on myopia. It contained a number of interesting facts. He said that myopia, or short-sightedness, had justly been called a disease of civilization, and unless prompt measures were taken to counteract the injurious influences which led to the development of the disease, it must more and more be regarded as a disease of civilized life. The most dangerous period for myopia to set in, was from the ages of five to fifteen years, and an examination of the pupils attending the schools of New York has led to the following discoveries: Out of 203 scholars attending the Thirteenth Street Grammar School only 6 were near-sighted. At Grammar School No. 58, 608 children were examined, of whom 8½ per cent. were suffering from myopia. This included 425 American children, among whom there were 34 cases of myopia, and 273 Germans, of whom 26 were suffering from myopia. At Grammar School No. 35, of 630 Americans, 10 per cent. were myopic, and of 266 Germans, 17½ per cent. were afflicted with the disease. At Columbia College 201 students were examined, and of these, 69, or 35 per cent. were found to be nearsighted; the percentage being greater in the academical department than in the school of mines. Further investigation, with a view to testing the hereditary nature of the disease, showed that of 45 Jews 40 per cent.

came from myopic families; of 82 German myopics, 29, or 35 per cent. came from myopic families; and of 160 American children only 49, or 31 per cent. had myopia in their families. In all cases it was found that myopia increased with the length of school life. Very serious complications arose in this disease by neglecting the use of glasses, and frequently total blindness resulted from this neglect.

Dr. Miltendorf especially insisted upon the early use of glasses. Perhaps it would be of still more importance to insist upon children being supplied with the right kind of disks, with sufficient light and good print. A model of the type to be used in school-books has been given by Dr. Javal, of Paris, and is reproduced by the Commissioner of education.

The type in which this is printed is as close an imitation of that specimen as can be given.

Dr. Javal thinks that "leading" does not much improve legibility, but that spacing letters does.

At the Practitioners' Society, which met this month, Dr. F. P. Kinnicutt read a very interesting paper on the "Oil of wintergreen as an efficient salicylate in acute rheumatism." The oil of wintergreen (oleum gaultheriæ) is a neutral salicylate acid, and a terebinthinate. It is given in doses of ten to fifteen minims, every two hours at first, in cases of acute articular rheumatism, just as in the salicylates.

Dr. Kinnicutt reported twelve cases, in all of which the fever and pains rapidly subsided, and the disease was apparently checked by his use of this oil. The effects seemed to be a little better than those gotten from the artificial salicylates. That is to say, there were no toxic symptoms, no cardiac depression, and no albumen in the urine. Another advantage was that the oil is agreeable to the taste, and does not disturb the stomach, as a rule. It was given floating on some menstruum, or in capsules with magnesia. In the last case, the drug is apt to nauseate, if given continuously in maximum doses.

The oil is very quickly eliminated, appearing in the urine within an hour. Furthermore, it is in quantity large enough to preserve the urine from decomposing for several days. It is suggested that it may be useful in cystitis. I believe that it has been tried for that purpose in Glasgow, and with some success.

Dr. T. A. McBride said that he had used the oil in muscular rheumatism, lumbago, etc., with great satisfaction. He had previously been accustomed to use muriate of ammonia and tincture of iron, but then wintergreen seemed to act better. I have tried it in a case of subacute rheumatism, following an acute headache, also in a case of lumbago. It certainly appeared to produce much relief.

Dr. George F. Shrady reported a unique case of successful tracheotomy in an infant eleven months old, suffering from diphteria. The disease was unmistakable, and the child was apparently dying when the operation was performed. There has been no previous successful case of tracheotomy in so young a child in this country, though I believe that some have been reported as occurring in Europe.

The National Association for the Protection of the Insane and Prevention of Insanity, about which I occasionally inflict news upon you, continues to survive its name, and is even developing an exuberant vitality. A meeting to discuss its work, is soon to be held in Philadelphia, I am told. The association has appointed corresponding members in all the States, and intends to embody its work in a semi-annual bulletin. There are 100,000 insane in the United States, and not half of them have asylum accomodation. Asylum management itself is generally under the ban of political machinery, or of a stupid conservatism. The Associa- of Asylum Superintendents does not represent any progressive or scientific work, although it is somewhat arousing itself as a result of recent agitation. The National Association has a field for work, therefore, in attacking political charities, and in encouraging a more general study of psychiatry.

A very interesting meeting of the Academy of Medicine took place this week, when Dr. T. E. Satterthwaite read a paper upon the "Etiology and Natural History of Tuberculosis." The reader gave a summary of the various facts bearing upon the morbid appearances, imfectiousness, parasitic origin, etc., of tuberculosis.

Some of his most important conclusions were as follows:

1. Tuberculosis is a disease that is hereditary, statistics having abundantly proved that there resides in certain families a greater or less susceptibility to the disease, which usually manifests itself in the pulmonary organs.

2. The most distinguishing characteristic of tubercle, is the occurence in the tissues, of minute, bright, glistening, translucent particles, that have been called miliary tubercles, granula, granulations, etc.

3. They are the result of an inflammatory process, because they can be produced by the introduction of mechanical irritants into the system.

4. When these minute bodies coalesce to form larger bodies and undergo a change of color, they are known as crude or yellow tubercles.

5. Some of them contain the reticulated tissue that is called adenoid; others do not, but contain indifferent cells, fibrillar tissue, etc.

6. In their gradual development, they undergo caseous change. More rarely they become cretaceous, or organized, when a cure may result.

7. Tubercles, after a time, excite inflammation *in situ*, which inflammation may be classed as a pneumonia (catarrhal, or desquamative).

8. Tuberculosis is inoculable and it "breeds true," always producing its kind if it produces anything, but other substances will also in a certain number of cases produce the same results; in fact, any organic substance that is capable of physical deterioration. It has not been satisfactorily proved that tuberculosis has a specific virus.

9. There is some good evidence going to prove that tubercle is contagious, *i. e.*, that it is capable of propagation by cohabitation, or, in other words, close association with persons that have the disease. The number of well-authenticated instances in the human being where the disease appears to have been spread in this way, indicates something more than a coincidence. This relation has been shown with a reasonable amount of certainty in horned cattle.

10. And yet bovine tuberculosis differs in some important particulars from the disease in men and the lower animals, though it is highly probable that the differences, which are mainly in the pathological appearance, are referable to differences in anatomical structure in the different animals.

11. From a sanitary standpoint, we should discountenance the use of milk or meat from phthisical cows, since if not absolutely infective, they are at least deficient in the proper nutritive elements, and for this reason alone should be excluded from the markets.

12. Though it does not appear that it can be shown that any person has ever been rendered tuberculous through the medium of bovine virus, still when the cattle used for vaccine purposes are slaughtered, those in charge of the farms should examine the viscera of all such cattle before the virus is furnished to physicians, and it should be specially understood that such an examination has been made in every instance where the virus is sold.

13. The parasitic theories of Klebs, Koch, and others, are still *sub judice*.

14. Tuberculosis and pulmonary phthisis are almost in all cases the same disease.

In the debate which followed, Dr. Francis Delafield gave a sketch of his view of tuberculosis. Miliary tubercules and tuberculous tissue were not, he said, always indentical Tuberculous tissue existed sometimes in the form of miliary nodules, sometimes diffused. In pulmonary phthisis, there was always an involvement of many different tissues, there were various inflammations and their products, all of which tended to obscure the tubercular proccss. He believed, however, that a tubercular process always accompanied phthisical processes in adults, and was essential to it. In other words, phthisis is a tuberculous disease. Dr. Robert Welch agreed with Dr. Delafield's views as to the unity of phthisis.

Referring to Koch's discovery of the *bacillus*, he said he had been uniformly successful in finding the parasite in phthisical lungs and sputa. He employed Ehrlich's method of staining. Dr. Welch seemed much inclined to credit Koch's views, and advanced several arguments in its support, these being chiefly from analogies to syphilis.

Dr. A. Jacobi could not explain the clinical facts of phthisis, except by assuming that it was not always the same as tuberculosis. He referred to the possibility of local inflammations and suppurations in children being the cause of a subsequent development of phthisis. He thought that the physician ought to watch and treat cases of measles, whooping-cough, abscesses, pneumonias, eczemas, etc., lest they become in time infective foci.

Among the three prominent pathologists who spoke, there was an entire unanimity as to the unity of phthisis and tuberculosis. The views of Niemeyer, said Dr. Satterthwaite, were based on facts which were not true. They have received a wider acceptance in this country than in Germany, according to Dr. Welsh. We now go back again to the views of Louis and Laennec.

A growing feature of some of our larger medical gatherings is the presence of women physicians. The lady doctor seems to be occupying a larger space in the public attention, if not affection, every year. A curious fact is the way in which novelists are seizing upon this class as material for imaginative studies. Howells causes "Dr. Breen" to attitudinize before his fancy, while he makes graceful comments upon her. Miss Phelps treats with more seriousness "Dr. Zay;" a German writer has taken the same subject for an elaborately philosophical romance. There is one common element in all these works of fiction, viz: the lady physician, on or about the last page, throws herself with unscientific abruptness into the arms of the admiring hero. I venture to state, from some knowledge of the lady physician, that her delineators know very little about her. The type of lady physician, at least, is not a fascinating one. The tendency of medical study is to rob woman of what charms she possessed, in the eyes, at least, of all young men. It is a curious fact, but a real one, that woman, as soon as she begins to work seriously for her livelihood, loses some of her attractiveness. There are many bright minds and noble characters among medical women, but that they offer good material for romance and love-stories, is certainly not true.

I have sometimes ventured to send you a few items "in lighter vein." It is rather discouraging to see them at once appropriated by other journals, and circulated without acknowledgment.

Herbert Spencer has just announced that the American is a failure (ethically), because he encroaches on other's rights too much, and does not defend his own enough. I beg you, therefore, to assault with your utmost editorial vigor any one who ventures to copy the following highly interesting case without acknowledging the trouble I have taken to get and copy its details. It was written by a clever surgeon of this city, Dr. Helmuth, and is, I think, rather good:

"A clever young fellow, a student at law,
Had an indolent swelling come under his jaw.
He took many drugs, still larger it grew,
Till it covered the jaw and the clavicle too.
And though it received every care and attention,
It much interfered with the act of prehension.
The pain was so great that the man became furious,
But relief came at last, when c. m. Mercurius
Arrested the sweating and slacken'd the thirst,
Then softened the swelling, which very soon burst;
But instead of 'the corn,' oh, strange to behold,
The tumor discharged quite a lump of pure gold,
Which a dentist had dropped in the gum, while essaying
To plug up a molar, long since decaying.
This morsel of gold, like an amœboid cell,
Worked its way to the surface; how, no one could tell,
But it shows how Dame Nature may sometimes grow bold,
Like most other women, and throw away gold.
The patient's delight can't be told in this stanza,
For he fancied his jaw had become a bonanza."

NEW YORK CITY, OCT. 21, 1882.

